# Evaluation of Sentinel-3A OLCI Products Derived Using the Case-2 Regional CoastColour Processor over the Baltic Sea

**DOI:** 10.3390/s19163609

**Published:** 2019-08-19

**Authors:** Dmytro Kyryliuk, Susanne Kratzer

**Affiliations:** Department of Ecology, Environment and Plant Sciences, Stockholm University, 10691 Stockholm, Sweden

**Keywords:** OLCI, Sentinel-3A, C2RCC-SNAP, validation handbook, Baltic Sea, high CDOM absorption

## Abstract

In this study, the Level-2 products of the Ocean and Land Colour Instrument (OLCI) data on Sentinel-3A are derived using the Case-2 Regional CoastColour (C2RCC) processor for the SentiNel Application Platform (SNAP) whilst adjusting the specific scatter of Total Suspended Matter (TSM) for the Baltic Sea in order to improve TSM retrieval. The remote sensing product “kd_z90max” (i.e., the depth of the water column from which 90% of the water-leaving irradiance are derived) from C2RCC-SNAP showed a good correlation with in situ Secchi depth (SD). Additionally, a regional in-water algorithm was applied to derive SD from the attenuation coefficient *K_d_*(489) using a local algorithm. Furthermore, a regional in-water relationship between particle scatter and bench turbidity was applied to generate turbidity from the remote sensing product “iop_bpart” (i.e., the scattering coefficient of marine particles at 443 nm). The spectral shape of the remote sensing reflectance (*R_rs_*) data extracted from match-up stations was evaluated against reflectance data measured in situ by a tethered Attenuation Coefficient Sensor (TACCS) radiometer. The L2 products were evaluated against in situ data from several dedicated validation campaigns (2016–2018) in the NW Baltic proper. All derived L2 in-water products were statistically compared to in situ data and the results were also compared to results for MERIS validation from the literature and the current S3 Level-2 Water (L2W) standard processor from EUMETSAT. The Chl-a product showed a substantial improvement (MNB 21%, RMSE 88%, APD 96%, *n* = 27) compared to concentrations derived from the Medium Resolution Imaging Spectrometer (MERIS), with a strong underestimation of higher values. TSM performed within an error comparable to MERIS data with a mean normalized bias (MNB) 25%, root-mean square error (RMSE) 73%, average absolute percentage difference (APD) 63% *n* = 23). Coloured Dissolved Organic Matter (CDOM) absorption retrieval has also improved substantially when using the product “iop_adg” (i.e., the sum of organic detritus and Gelbstoff absorption at 443 nm) as a proxy (MNB 8%, RMSE 56%, APD 54%, *n* = 18). The local SD (MNB 6%, RMSE 62%, APD 60%, *n* = 35) and turbidity (MNB 3%, RMSE 35%, APD 34%, *n* = 29) algorithms showed very good agreement with in situ data. We recommend the use of the SNAP C2RCC with regionally adjusted TSM-specific scatter for water product retrieval as well as the regional turbidity algorithm for Baltic Sea monitoring. Besides documenting the evaluation of the C2RCC processor, this paper may also act as a handbook on the validation of Ocean Colour data.

## 1. Introduction

In October 1978, the Coastal Zone Color Sensor (CZCS) was launched on the Nimbus 7 satellite. This first Ocean Colour mission was initially only thought as a proof of concept mission in order to test if it is possible to measure ocean productivity from Space. Although only designed to be operated for 1 year it measured successfully until June 1986. The first images from the CZCS revealed the true nature of seas and oceans which were shown to be much more complex and dynamic than point sampling had many made to believe [1]. The instrument was used, for example, to derive global productivity, and it improved our understanding of the significance of phytoplankton production which makes up about 50% of the total global production [2,3]. However, CZCS had problems in distinguishing between chlorophyll-a (Chl-a) and coloured dissolve organic matter (CDOM), and subsequent missions by the National Aeronautics and Space Administration (NASA) and the European Space Agency (ESA) were designed to progressively improve the retrieval of different water constituents from Space.

**Figure 1:**
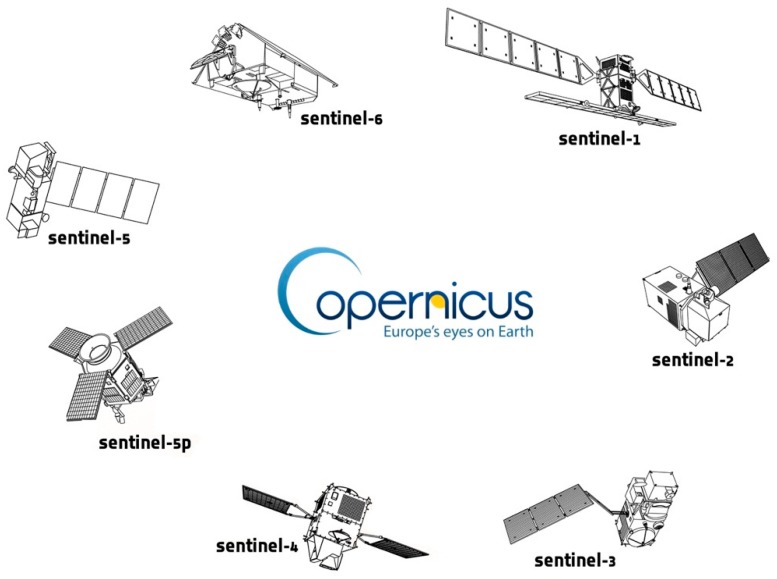
ESA’s operational Copernicus’s program with the Sentinel family. ©ESA (modified from: [7]). We investigate here data from OLCI on Sentinel-3 (S3) for Baltic Sea applications.

In 2014, the European Commission in partnership with ESA established the so-called Copernicus Programme (previously called Global Monitoring for Environment and Security, GMES). It is an operational programme that was designed to achieve accurate, near-real time information delivery to facilitate improved management of the environment and to understand and alleviate the effect of climate change [4,5]. ESA has been developing a new constellation of satellites called Sentinels for the operational needs of the Copernicus program. Each mission consists of at least two satellites to facilitate increased coverage and provide robust datasets for Copernicus Services. These satellite missions possess a range of sensing capabilities such as radar, multi-spectral imaging instruments for monitoring of land, oceans and the atmosphere (Figure 1; [6]).

### 1.1. Sentinel-3

The Sentinel-3 (S3) mission was designed to support ocean forecasting systems, environment and climate monitoring. Sentinel-3A and Sentinel-3B were launched on 16 February 2016 and on 25 April 2018, respectively [8]. The Ocean and Land Colour Instrument (OLCI) on Sentinel-3 has similar specifications as its predecessor Medium Resolution Imaging Spectrometer (MERIS) on ENVISAT with the ability to perform measurements of bio-optical constituents in coastal regions [9,10,11,12]. In the first half year after launch OLCI was going through a thorough calibration and commissioning phase. Products have become available to a broader scientific community in late October 2016. As mentioned before, Sentinel-3B, has joined S3A in orbit on 25 April 2018. The pairing of those two satellites is designed to optimize the coverage and data delivery to the users. The operational lifespan is expected to be 7 years with consumables for 12 consecutive years. There are plans to launch two additional Sentinel-3 satellites (S3C and S3D) with a total expected mission time of approximately until the end of 2030. In total, S3 houses seven sensors for measuring the ocean’s properties. These include instruments for measuring Ocean Colour, sea surface temperature (SST), radar and microwaves, Doppler positioning, a laser reflector and ESA’s global Navigation Satellite System (GNSS). There are several thematic areas and services that the mission aims to provide: Numerical Ocean Prediction, Maritime Safety and Security, Open Ocean and Ice Monitoring, Atmospheric services, Global Land Monitoring Application, etc. [13]. Of main interest for the Ocean Colour community are the ocean colour measurements performed by OLCI with the main focus on coastal zone monitoring, i.e., observing and monitoring the environmental status and the characteristics of coastal zones in order to support environmental monitoring of water quality and the development of seasonal phenomena (such as harmful algal blooms), and to improve management of coastal habitats [13].

The OLCI bands (Table 1) are a heritage from MERIS and complemented to optimize the measurement of Ocean Colour over the open sea and the coastal zone. An additional band at 1.02 µm was added to improve atmospheric correction, as well as a channel at 673 nm (7.5 nm wide) for improved Chlorophyll fluorescence measurements. The swath width of the OLCI instrument is 1270 km and the instrument is tilted across track by 12.6° in the opposite direction to the sun in order to minimize potential impact of sun glint [14].

### 1.2. OLCI Products Are Available on Three Main Levels

Level-1 (L1) products contain radiance for each pixel in the instrumental grid. Level-1b contains calibrated, orthogeolocated and spatially resampled Top-of-Atmosphere (TOA) radiances for all spectral bands [15]. Level-2 Water (L2W) products come in two versions, OL_2_WRR and OL_2_WFR that are outputs from the OLCI Level-2 processor that derives water and geophysical products at Reduced (~1 km/pixel) and Full (300 m/pixel) resolution. L2W products are the ones that are most relevant and useful for monitoring of coastal areas, since they provide bands with geophysical and bio-optical constituents expressed in actual concentrations [16] or in terms of inherent optical properties (IOP’s), i.e., absorption and scattering properties. Additionally, there are several third-party processors capable of deriving remote sensing reflectance and water quality variables from OLCI L1 data [17,18]. The focus of this study is on the assessment of the Case-2 Regional CoastColour processor provided by Brockman Consult (Hamburg, Germany) and its applicability in Baltic Sea waters.

### 1.3. The Case-2 Regional CoastColour Processor

The Case-2 Regional CoastColour (C2RCC) processor v1.0 is a software for processing Ocean Colour data from different satellite instruments (OLI, MERIS, MODIS, SeaWiFS, MSI and OLCI) [18]. The processor is a further development of the Case-2 Regional Processor (C2R) produced by Doerffer et al. [19], which was further modified during the CoastColour project (www.coastcolour.org). The main concept is based on radiative transfer modelling of water using radiative transfer theory (based on Hydrolight [20]) and the vector successive order of scattering (SOS) atmospheric model with aerosol optical properties derived from NASA AERONET-OC measurements [21,22]. Models are parameterized with an elaborate bio-optical ocean-atmosphere model using a large database of water-leaving reflectances and relating the optical properties from various coastal areas around the world. The calculations are done for all the spectral bands of a given sensor. This is considered an important aspect as it implies consistent modelling throughout all sensors [18], making the approach generic across missions. 

Processing is done by a set of neural networks that are generated for each sensor using a subset of bands of that sensor. The main neural net derives water-leaving reflectance ‘*Rw*’ after atmospheric correction. Subsequently, the ‘IOP’ net retrieves the absorption and scattering coefficients from the water-leaving reflectance. Additional information on processing is given in more detail in Brockmann et al. [18].

Apart from being applicable to a wide range of satellite sensors, the C2RCC provides flexibility in adjusting ancillary parameters that can be adapted by the user to the specific inherent optical properties (sIOP’s) as well as ancillary parameters of the local waterbody. The ancillary parameters include e.g., salinity, temperature, ozone, air pressure as well as the specific IOP’s, namely the Chl-specific absorption coefficient and the specific scatter of Total Suspended Matter (TSM) at 442 nm. Thus, the approach allows for adjustment of local relationship between IOP’s and concentrations of optical substances in the water that are regionally variable [18]. The Chl-specific absorption coefficient for the Baltic Sea did not show any significant difference when compared to other seas and oceans [23], while the TSM-specific scatter was found to be 1.016 ± 0.326 m^2^g^−1^ (*n* = 56) which is substantially higher than the value assumed, for example, in the MERIS (MEGS 8.4) processor (0.578 m^2^g^−1^) [23]. The TSM-specific scatter allows to derive the TSM concentration from scatter which in turn is derived from the reflectance measured by the MERIS or OLCI sensors, respectively.

It is the aim of this study to apply the C2RCC-SNAP with regionally adjusted TSM-specific scatter and to evaluated the retrieval of the L2 remote sensing reflectance, *R_rs_*. Additionally, the L2 C2RCC-SNAP water products “conc_chl” (chlorophyll-a, Chl-a), “conc_tsm” (Total Suspended Matter, TSM), “iop_adg” (i.e., the sum of organic detritus and Gelbstoff absorption at 443 nm) and “iop_agelb” (i.e., the absorption coefficient of Gelbstoff at 443 nm) using Coloured Dissolved Organic Matter (CDOM) as a proxy, as well as the Secchi depth using (I) the product “kd_z90max” (i.e., the depth of the water column from which 90% of the water-leaving irradiance are derived; corresponding to 1/*K_d_*_min) as a proxy as well as by (II) applying a regional Secchi depth algorithm developed by Alikas et al. [24] to the *K_d_*(489) product generated by C2RCC-SNAP. 

Additionally, we aim to develop and test a regional algorithm from optical in situ data for predicting turbidity from the OLCI product “iop_bpart” (i.e., the scattering coefficient of marine particles at 443 nm) *R_rs_*. For this, we use data from the NW Baltic proper as well as the Gulf of Bothnia, spanning over a large range of CDOM absorption and TSM concentrations.

A more general aim of this paper is to share our experience with hands-on validation of Ocean Colour satellite data, dating back to the late 90 ties. In this sense, this article can be used as a hand-book for the validation of Ocean Colour data.

## 2. Materials and Methods

### 2.1. Area of Investigation

**Figure 2:**
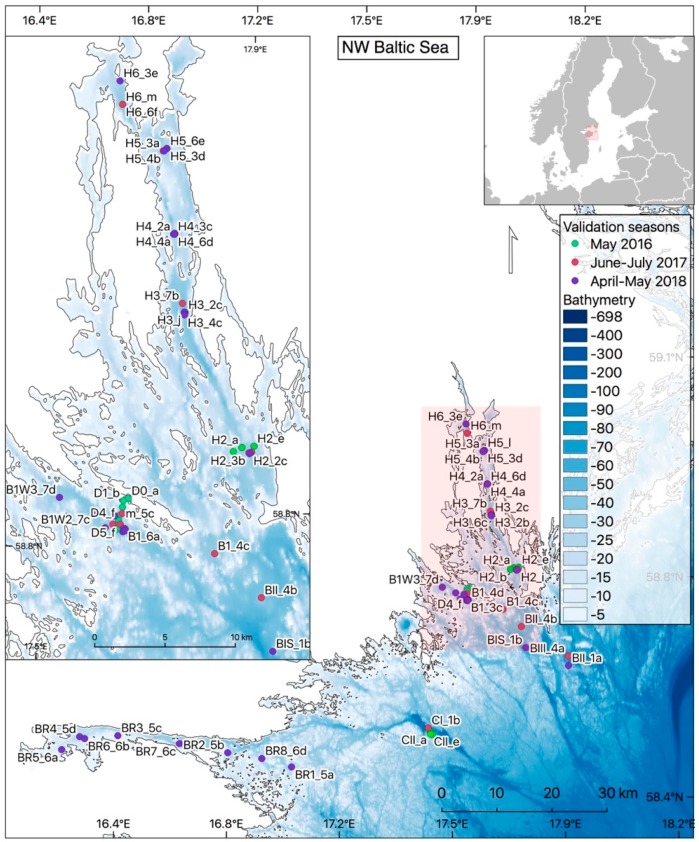
Location of in-situ sampling stations used for validation of Sentinel-3A during dedicated sampling campaigns in May 2016, June–July 2017 and April–May 2018. Bathymetry data [27]; HELCOM sub-basins [28]. European coastline shapefile (European Environment Agency, [29]). Country boundaries (Natural Earth, [30]).

Himmerfjärden (HF) bay, located in the NW Baltic proper, is one of the most frequently monitored waterbodies in the Baltic Sea area (Figure 2). The narrow bay is situated at about 60 km south of Stockholm and is divided into several sub-basins, each separated by intersecting sills with a mean and maximum depth of 17 and 52 m, respectively [25]. HF bay connects to Lake Mälaren in Södertälje in the northern part of the bay via a lock. The HF Sewage Treatment Plant is also located in the inner bay (close to station H5 in Figure 2). The bay has been extensively monitored since the 70 s with five coastal monitoring stations inside HF bay (H2, H3, H4, H5 and H6) and a coastal reference stations–B1 about 4 km SW of Askö as well as a deep-water reference station (BY31) which is situated at about 30 km off-shore (note that BY31 is not shown in Figure 2). These stations are part of the Himmerfjärden Eutrophication Study [26] and form part of the Swedish coastal monitoring program.

### 2.2. In Situ Sampling 

Optical water quality variables, i.e., absorption and attenuations measurements (WetLabs AC9) as well as the spectral attenuation coefficient, *K_d_*(490) —estimated from radiometric system TACCS, Satlantic Inc. (Halifax, NS, Canada)—were measured during dedicated sea-truthing campaigns in HF bay and adjacent areas using Askö Laboratory as a base. The optical cruises took place during several campaigns during 2016–2018, using various small boats and research vessels and spanning over the main vegetation periods and including periods with both high and low phytoplankton biomass (Figure 2). On 9 May 2016, an additional mini-transect with a reduced number of optical variables (Secchi depth, TSM and turbidity) was performed between the island of Askö and B1 (denoted station D in Figure 2) using a small motor boat in order to evaluate small-scale variability in the coastal zone. Complementary samples were taken by the Swedish coastal monitoring program from Stockholm University performing a planned transect along the coastal gradient in HF during the 9 May 2016 (stations B1, H2–H6). The 9th of May also coincided with one of the first (cloud-free) satellite image taken by Sentinel-3A over HF. During 2017, an additional dedicated optical campaign was organized by the marine remote sensing group at Stockholm University between 13 July and 22 Aug 2017 with additional samples taken by the coastal monitoring group at Stockholm University. The optical campaign was designed to capture the summer bloom variability in HF. A similar campaign was organized in 2018, between 13 April and 5 May in order to follow the development of the spring bloom. Additionally, a two-day transect was performed during 24–25 April 2018 through Bråviken bay using the R./V. Electra af Askö belonging to Stockholm University (stations BR in Figure 2). This transect was chosen on the basis of regional satellite images, showing a more optically explicit gradient and water quality ranges not found in HF bay [31,32]. This particular transect was not included in the following match-up analysis due to 100% cloud cover of the area during sampling. However, the data was included in the analysis that aims to derive a regional algorithm between turbidity and particle scatter at 440 nm (b_440_), Additionally, three short cruises (4 days long each) were performed across the Gulf of Bothnia during May, June and July 2018 in order to extend the range of values for deriving the turbidity vs. particle scatter algorithm and to make it applicable on a regional scale. The campaigns in the Baltic proper were planned in concordance and chosen on the basis of scheduled Sentinel-3A overpassed predicted using the ‘Earth observation Swath and Orbit Visualization tool’ (ESOV v2.3-4; [33]). The campaigns in the Gulf of Bothnia were planned and performed by the monitoring group of Umeå University based in Nordby in collaboration with the Swedish Coast Guards (Kustbevakningen) on R./V. KBV181. In this paper we use turbidity data (Hach Lange GmbH, Düsseldorf, Germany) and the scatter derived from the AC9 [23] from these cruises in the Gulf of Bothnia in order to extend the range of these parameters for algorithm development, making the case for a regional rather than a local algorithm.

Additional screening for cloud-free dates was done closer to the actual ‘overpass’ time of the satellite using the weather App ‘Weather Pro’ with 7-day forecasting. This was done in order to avoid cloud interference that may result in low-quality match-ups, or no match-ups at all. As match-up we define here a match-up in space and in time of both in situ and satellite measurement data (measured within ± 3 h of the overpass). The sampling stations were designed to form transects either starting at the head of HF bay (either at H6 or H5), moving southwards towards the open sea, or in opposite direction, dependent on the cloud and weather conditions. As it takes approximately 3 h to travel from Askö to H6 (Figure 2) one needs to account for about 7 h to perform a transect of four stations with 40 min required to perform all optical measurements and approximately 20 min sailing between each station. The idea of doing optical measurements along the coastal gradient was to capture the variability of optical properties and to achieve a wide range of each optical in-water variable. 

**Figure 3:**
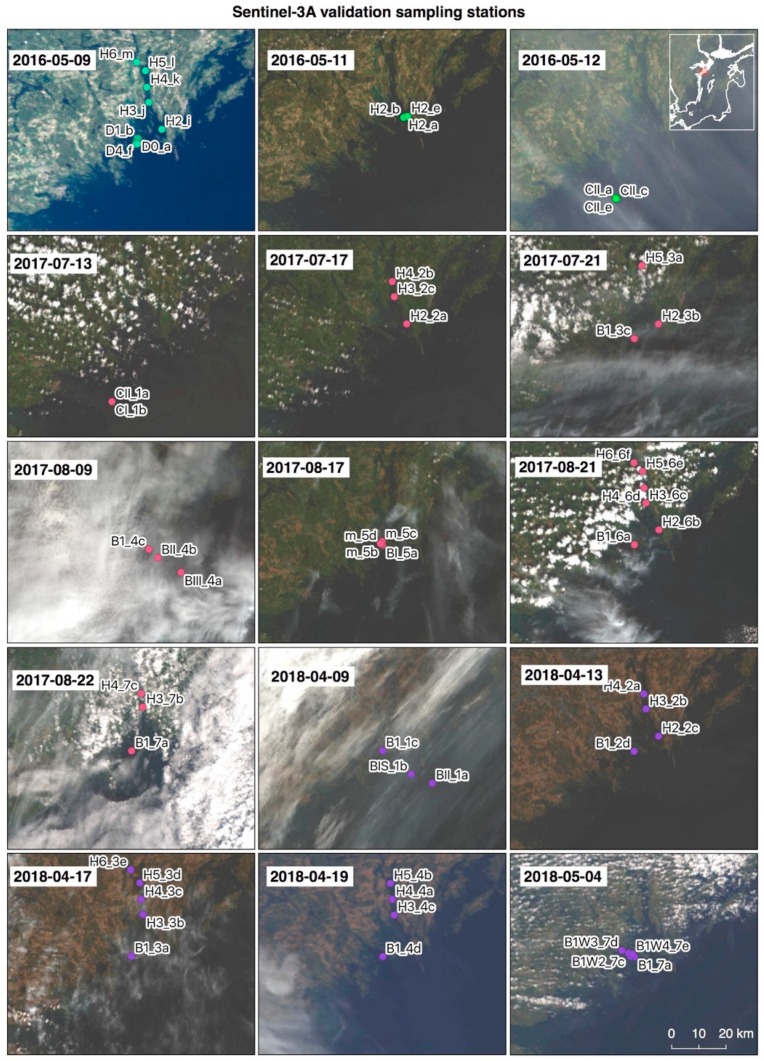
Overview of sampling stations during dedicated Sentinel-3A OLCI validation campaigns in the Baltic proper during May 2016, July–August 2017 and April–May 2018 super-imposed on full resolution True Color satellite images of the stations sampled in Baltic Sea coastal waters. The main challenge associated with validation efforts in the Baltic Sea is the extensive cloud cover as can be seen here on several satellite images. About 50% of the match-up stations were flagged.

The weather condition and cloud cover were generally quite difficult to predict on the planned days of overpasses. On several occasions it was decided to take water samples and optical measurements despite cloud cover which generally means that there is no matching satellite data available. Although not useful for validation, the optical data can still be used for algorithm development. The overview of True Color (red-blue-green bands) satellite images acquired during planned sampling campaigns regardless of weather conditions are presented in Figure 3; an additional overview of match-up stations is shown in Table 2. 

### 2.3. Optical In-Situ Data

Absorption and scattering were measured in situ using an AC9^+^ system (WetLabs, Philomath, OR, USA) with a 20 cm pathlength and fitted with a CTD (STD, SAIV-AS, Laksevag, Norway) to derive salinity from conductivity and to measure water temperature. After taking the measurements the data was transferred from binary to engineering units using a custom Excel program (Kratzer et al. [23]) which also applies the WetLabs calibration file (device file). A total number of 64 AC9 profiles were measured in the Baltic proper and in the Gulf of Bothnia combined during 2017–2018. For the validation of Sentinel-3A OLCI reflectance data a tethered Attenuation Coefficient Sensor (TACCS, Satlantic Inc., Halifax, NS, Canada) was used at the sea surface. The TACCS is a multi-channel radiometer that includes seven channels for upwelling radiance, *L_u_* (412, 443, 490, 510, 560, 620 and 670 nm) and three downwelling irradiance (*E_d_*) channels (443, 491 and 670 nm) that has been successfully used for the previous validation of MEdium Resolution Imaging Spectrometer (MERIS) data in the Baltic Sea coastal areas [34,35,36]. The reflectance data was derived from TACCS and AC9 data combined using a custom-made processor [23,37]. The output of the TACCS processor is water-leaving reflectance, rhow. The latter was then converted to remote sensing reflectance, *Rrs* (i.e., by multiplying rhow by the factor π) in order to compare the spectral reflectance to the remote sensing reflectance derived from S3 data.

For the validation of water products, the water samples were collected at about 15–20 cm below the sea surface using a dedicated durable sampling bucket and further processing was done according to the optical protocols detailed in [23]. Both the organic and inorganic fraction of Total Suspended Matter (TSM)—also termed Suspended Particulate Matter (SPM)-was measured using the gravimetric method detailed by Strickland et al. [38] and in the MERIS protocols [11]. Chlorophyll-a (Chl-a) was processed according to Parsons et al. [39] and Jeffrey et al. [40]. The samples were kept in liquid nitrogen until analysis (within 2 months) and were extracted using a sonicator. Coloured Dissolved Organic Matter (CDOM) was measured spectrophotometrically [41,42] after filtration through 0.22 μm membrane filters. Secchi depth was measured by lowering a 30 cm disk (in diameter) into the water and noting the depth at which the disk disappeared from the observer’s view. Turbidity was measured using a portable bench turbidity meter (Hach Lange 2100Qis) calibrated against a standard formazin solution provided by the manufacturer and given in Formazin Nephelometric Unit (FNU) as the instrument measures at 860 nm. The samples were carefully mixed before each measurement as described in [43].

### 2.4. Satellite Data Processing

For the match-up analysis Level-1b data IPF Processing Baseline 2.23 was downloaded from Copernicus Online Data Access (CODA; REProcessed, [44]) for the period 26 April 2016–29 November 2017 and from the CODA portal [45] after 30 November 2017 [46]. All data were processed by the same Level-1 processor. Further, all data were downloaded from descending orbits, full-resolution (FR 300 m/pixel), mode “Non Time Critical” and carry product type name “OL_1_EFR_” following ESA’s naming convention [47], (Table 3).

The 15 match-up scenes (Level-1 OL_1_EF_ products) were further processed using ESA’s SentiNel Application Platform (SNAP v6.0 [48]). Level-2 processing was done via the Thematic Water Processing in SNAP using the C2RCC v1.0 OLCI processor (version 1 nets-2016) without vicarious calibration. A set of Processing Parameters (Table 4) which can be freely defined via a user interface was applied, thus adjusting the processor to the approximate mean conditions and optical properties of the Northwestern Baltic Sea encountered during each sea-truthing campaign. The list of the parameters which are user-defined are listed in Table 4 and parameters which were adjusted to local and seasonal water type are highlighted in bold. The remaining parameters were set to the default values and thus are the same as used in ESA’s ground segment processing of L2W products available through EUMETSAT [18]. The ozone values and the barometric pressure were also set to the default values (330 Dobson; 1000 mBar). Additionally, the box called ‘Use ECMWF aux data of source product’ was also ’ticked’ (On); it is applied in order to retrieve these values from L1b data if available. 

The L2 products generated using the C2RCC and a subset of products listed in bold (Table 5) are further used for comparison to in situ values. Pin-pixels corresponding to the sampling stations (Figure 2) were extracted using a 1 × 1 pixel window and invalid pixel-data were filtered out using C2RCC processor-specific flags. A set of valid-pixel expressions were tested and evaluated and the most appropriate flag expression was identified: ‘c2rcc_flags.Valid_PE && !c2rcc_flags.Cloud_risk && ! c2rcc_flags.Rhow_OOS && !c2rcc_flags.Rtosa_OSS’. The Cloud_risk flag denoting ‘high downwelling transmission indicates cloudy conditions’, Rhow_OSS flag means that the reflectance (Rhow) input spectrum to derive IOP’s from the neural net is probably not within the training range of the neural net, and that the inversion thus is likely to be wrong and Rtosa_OSS denotes that the input spectrum to the atmospheric correction neural net is out of the training range of the processor.

### 2.5. Turbidity Algorithm Development

Using a historical data set from 2000–2010 a prototype turbidity algorithm had been developed using optical data from field work in the NW Baltic Sea during 2001–2010 based on linear regression analysis:

*t* = 0.99 × *b*_440_ + 0.24; *R*^2^ = 0.56; *n* = 71 (algorithm Turb1 in Table 5). (1)

For this algorithm turbidity had been generated from TSM measurements using the algorithm developed by Kari et al. [43].

The relationship between actual turbidity and particle scatter *b*_440_ derived from this study (2016–2018) using regional data including the Gulf of Bothnia showed a higher correlation (*r* = 0.79; *n* = 64) but a very similar regression algorithm:

*t* = 1.02 × *b*_440_ + 0.18; *R*^2^ = 0.62; *n* = 64 (2)

The algorithm between ln (turbidity, *t*) and ln (b_440_), the particle scatter at 440 nm, was:

ln *(t)* = 0.82 ln (*b*_440_) + 0.14; *R*^2^ = 0.62; *n* = 64 (algorithm Turb2 in Table 5). (3)

Turb1 was used in the final evaluation as it was gathered from an independent (by location and time) data set from 2000–2010 and was still sufficiently close to the regional algorithm.

### 2.6. Testing of Various Secchi Depth Algorithms

For estimating Secchi depth (SD) the C2RCC-SNAP product “kd_z90max” was used along with the regional Secchi depth algorithm provided by Alikas et al. [24,49] which was found to retrieve the most reliable results when tested against the new validation data set (2016–2018), and was therefore used in this study to generate Secchi depth from OLCI *Kd*(489) data (i.e., the irradiance attenuation coefficient at 489 nm) along with “kd_z90max” data from C2RCC-SNAP. 

### 2.7. Statistical Evaluation of Match-up Data

Further, each individual remote sensing value (pixel value) covering the station position was directly compared to the corresponding in situ measurement (i.e., match-up data). The L2 C2RCC-SNAP products used in the match-up analysis were the Chl-a product “conc_chl” which was evaluated against the Chl-a concentration (mgm^−3^)) measured in situ, the TSM product “conc_tsm” evaluated against Total Suspended Matter (TSM (gm^−3^)), “iop_agelb” and “iop_adg” products against the absorption of Coloured Dissolved Organic Matter (CDOM), ($m^{-1}$)), and “kd_z90max” against Secchi depth, SD (m), along with SD derived from Alikas et al. [24] and the products Turb1 and Turb2 (Table 5) against bench turbidity [FNU] measured in situ. 

The discrepancies between matching in situ and OLCI data were evaluated using the Mean Normalized Bias (MNB), indicating the off-set from the 1:1 line, the Root Mean Square Error (RMSE)—a measure of the relative error (scatter), and the average of Absolute Percentage Difference (APD)—indicating the overall error [50,51]:

(4) $$\left(\mathrm{MNB}\right)\;\left(\%\right)=\frac{1}{N}\overset{N}{\underset{i=1}{\sum }}\left(\frac{{\mathrm{OLCI}}_{i}-{\mathrm{In}\;\mathrm{situ}}_{i}}{{\mathrm{In}\;\mathrm{situ}}_{i}}\right)\;\times \;100$$

(5) $$\left(\mathrm{RMSE}\right)\left(\%\right)=\;\sqrt{\frac{1}{N}\overset{N}{\underset{i=1}{\sum }}{\left(\frac{{\mathrm{OLCI}}_{i}-{\mathrm{In}\;\mathrm{situ}}_{i}}{{\mathrm{In}\;\mathrm{situ}}_{i}}\right)}^{2}}\;\times \;100$$

(6) $$\left(\mathrm{APD}\right)\left(\%\right)=\exp\left(\mathrm{mean}\left|\ln\left(\frac{{\mathrm{OLCI}}_{i}-{\;\mathrm{In}\;\mathrm{situ}}_{i}}{{\mathrm{In}\;\mathrm{situ}}_{i}}\right)\right|\right)-1\;\times \;100$$

as well as the Spearman’s correlation coefficient, *ρ* (which does not assume normal distribution of the data). The results and the final graphs were produced using *R* (v3.6.0; {ggpubr}package v0.2). The OLCI L2 remote sensing reflectance was also extracted for each match-up and its spectral shape (400–700 nm) was evaluated against in situ reflectance spectra measured in the Baltic proper by the TACCS radiometer.

## 3. Results

The WeatherPro App for 7-day forecasting showed to be rather reliable making it possible to plan the field campaigns closer to the predicted satellite overpass. The measurements in Bråviken coincided with the launch of S3B. Unfortunately, the conditions were cloudy. However, as this area had not been sampled by the marine monitoring group before the optical measurements were performed despite the lack of possible match-ups. The data may be regarded as especially valuable from an optical point of view as this bay has shown an especially large range of optical properties for the NW Baltic Sea, and the Swedish coast in general [31,32], and thus was also included in the turbidity algorithm development.

### 3.1. Remote Sensing Reflectance C2RCC-SNAP

**Figure 4:**
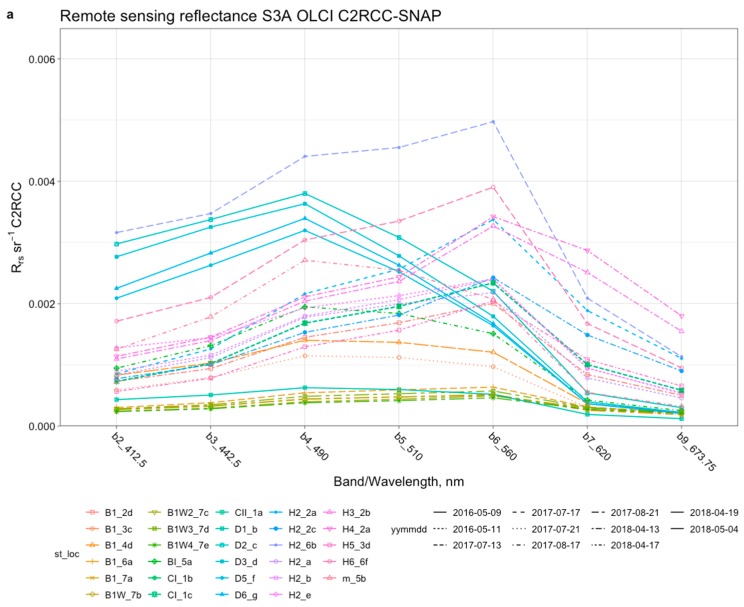

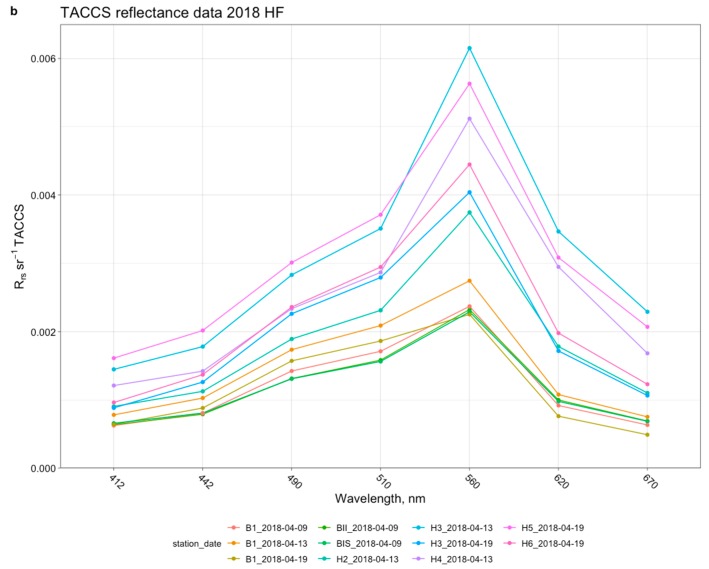
(**a**) Remote sensing reflectance *R_rs_* derived from S3A OLCI FR using C2RCC processor plotted in the 400–673 nm range for all sampling stations from all three validation campaign seasons in HF, 2016–2018; (**b**). Remote sensing reflectance, *Rrs*, derived from the TACCS processor during the field campaigns in the Baltic proper 2018. H and B stations denote stations in the Baltic proper. The in situ reflectance data does not indicate the observed shift towards 490 nm as indicated by the satellite data.

Remote sensing reflectance, *R_rs_*, values derived for sampling stations measured by Sentinel-3A OLCI using the C2RCC-SNAP were plotted for all bands and stations that passed the valid-pixel expression, thus indicating valid pixels (Figure 4). 

*R_rs_* has been derived for 34 sampling stations spanning for dates starting 9 May 2016 and ending 4 May 2018. *R_rs_* showed two sets of reflectance spectra with two distinct peaks—explicitly at 560 nm (for the majority of the stations) and a less obvious peak at 490 nm (Figure 4a) for some of the stations. The peak at 490 nm is characteristic for clear ocean water (or so-called optical Case-1 waters) and was here observed for stations D1_b, D2_c, D3_d, D5_f, D6_g, B1_3c, B1_4d, B1_h and BI_5a. Figure 4b) shows the remote sensing reflectance derived in situ from the TACCS measurements during field campaigns in the Baltic proper during 2018. The in situ reflectance data does not indicate the observed peak at 490 nm, as indicated by some of the satellite reflectance spectra. However, in some incidences (not shown in Figure 4), there was a slight shift towards the red part of the spectrum in the Gulf of Bothnia due to extremely high CDOM absorption (with maximum *a*_CDOM (440)_ values of 5.23 m^−1^).

### 3.2. Water Samples

The chlorophyll-a values ranged between 1.1 and 28.5 µgL^−1^ throughout all validation campaigns in the Baltic proper with mean concentrations of 5.9 µgL^−1^. The higher range values were measured in 2018 during the spring bloom with the highest Chlorophyll-a value of 28.58 µgL^−1^ measured in Himmerfjärden at station H4 on the 13 April 2018. Similarly, the highest value measured in the Bråviken transect was 25.05 µgL^−1^ measured on 24 April 2018 at station BR1, just outside Bråviken. The Chl-a values in May 2016 during an international intercalibration workshop were relatively low, ranging between 1.41–2.91 µgL^−1^ with a mean value of 2.01 µgL^−1^. During summer 2017, Chl-a ranged between 1.5–6.11 µgL^−1^ with a mean value of 3.4 µgL^−1^. These values compare well to previous values measured in the Baltic proper during summer [41,52]. In the Bothnian Sea there was still ice in the most northerly parts during the measurements in spring (14–17 May 2018). The chlorophyll-a values in the Gulf of Bothnia ranged between 0.55–8.91 µgL^−1^ with a mean value of 2.95 µgL^−1^ and a median of 2.53 µgL^−1^. The highest value was measured in May in near-coastal waters at the Swedish High Coast (Höga kusten) during the ice thawing and the spring flood.

Total Suspended Matter ranged in the Baltic proper between 0.34 and 4.9 gm^−3^ with a mean value of 1.6 gm^−3^. The highest values were observed in Bråviken in 2018 ranging from 2.26–4.91 gm^−3^ with a mean value of 3.76 gm^−3^. During the intercalibration workshop in May 2016 the values were relatively low, ranging from 0.38–2.00 gm^−3^ with a mean value of only 0.79 gm^−3^. Turbidity was measured between 0.27 and 5.0 FNU in the Baltic proper with a mean value of 1.2 FNU. In the Gulf of Bothnia turbidity ranged between 0.22–8.9 FNU with a mean value of 1.5 FNU and the highest values occurring in the Råne estuary during the ice-thawing period in May. Secchi depth was observed between 1.9 and 11 m in the Baltic proper with a mean value of 5.9 m. In the Gulf of Bothnia, it ranged between 0.7 and 8 m with a mean value of 3.64 m. The absorption of Coloured Dissolved Organic Matter, a_CDOM440_, ranged between 0.2 and 1.6 m^−1^ in the Baltic proper with a mean absorption of 0.53 m^−1^. The Bråviken transect showed a range of 0.4 and 1.62 m^−1^ with a mean absorption of 0.98 m^−1^. In the Gulf of Bothnia CDOM ranged between 0.45 and 5.23 m^−1^ with a mean of 1.62 m^−1^.

Particle scatter *b*_440_ derived from the AC9 attenuation and absorption measurements from the Baltic proper ranged from 0.43–3.37 m^−1^ with a mean of 1.52 m^−1^. Bråviken showed similar values with *b*_440_ ranging from 0.44–3.37 m^−1^ with a mean of 2.00 m^−1^. The Gulf of Bothnia showed a very low minimum value of 0.23 m^−1^ in the open sea and a maximum value of 2.77 m^−1^ and a mean value of 0.83 m^−1^.

### 3.3. Satellite-Derived Water Products in Relation to in Situ Water Samples

**Figure 5:**
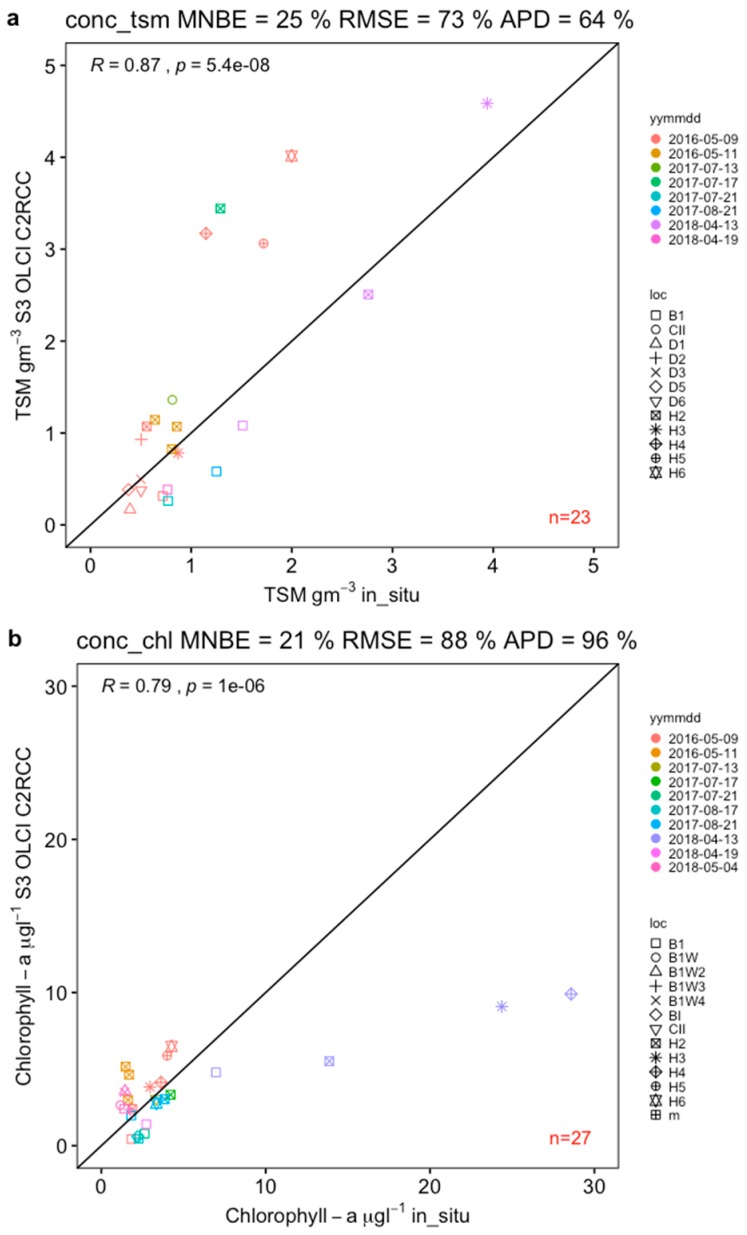
Concentration of (**a**) Total Suspended Matter (conc_tsm) and (**b**) Chlorophyll-a (conc_chl) derived from S3A OLCI data using C2RCC plotted again in situ concentrations.

Total Suspended Matter was overestimated by C2RCC-SNAP by 25% MNB, 73% RMSE and 64% APD (Figure 5a), which is within similar error margins previously observed in MERIS validation campaigns in the HF area [36]. 

The substantial scatter of points (relative error) expressed as RMSE was mostly observed at higher ranges of values (above 2.0 gm^−3^). Most of the points in the lower range (below 2.0 gm^−3^ showed less scatter and were well aggregated along the 1:1 line). The Spearman’s rhow, *ρ* = 0.87 showed a high correlation between satellite and in situ measurement and was significant (*p* < 0.001) (Figure 5a).

Chlorophyll-a overestimation was slightly lower, with 21% MNB, 88% RMSE and 96% APD (Figure 5b). The majority of data points aggregated well along the 1:1 line below concentrations of 10 µgL^−1^. Higher Chl-a concentrations (>10 µgL^−1^) derived for the inner stations in HF bay (H4, H3 and H2; Figure 2), however, were strongly underestimated. 

**Figure 6:**
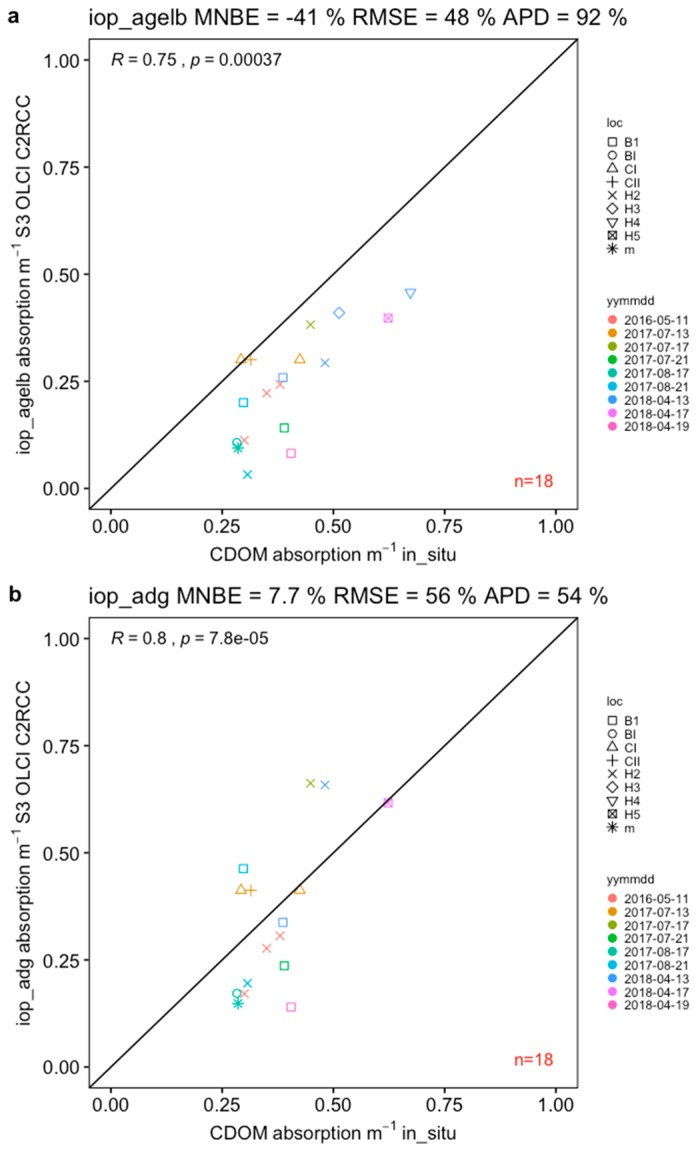
Absorption coefficient of (**a**) Gelbstoff at 443 nm (iop_agelb) and (**b**) detritus + gelbstoff absorption at 443 nm (iop_adg) derived from S3A OLCI using C2RCC-SNAP both compared to in situ absorption of CDOM, *a*_CDOM_ (440).

The C2RCC-SNAP retrieves several IOP’s that are related to CDOM measured in situ. The absorption coefficient of Gelbstoff at 443 nm (iop_agelb) and the combined product detrital + Gelbstoff absorption at 443 nm (iop_adg) was underestimated by −41% MNB, 48% RMSE and 92% APD, which was consistent with previous assessments of CDOM satellite products over the Baltic Sea, however with a slightly improved MNB (Figure 6). Interestingly, the product “iop_adg” compared better to CDOM absorption with overall improved statistics (7.7% MNB, 56% RMSE and 54% APD) even though the detrital absorption was not included in our validation campaigns. Overall, efforts to predict CDOM from remote sensing data are moving into the right direction. However, the results still confirm the challenges to derive CDOM and detrital absorption accurately in the Baltic Sea. 

**Figure 7:**
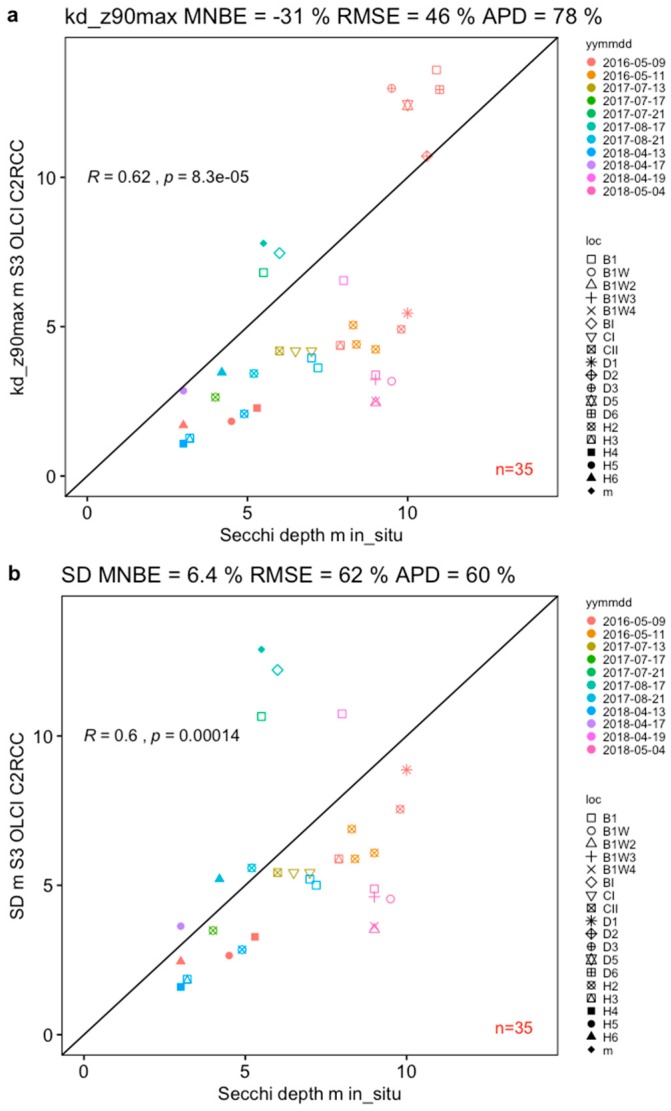
A proxy for Secchi depth (**a**) “kd_z90max”, and (**b**) the Secchi depth algorithm based on *Kd*(489) (diffuse attenuation coefficient at 489 nm) from Alikas et al. [24] derived from S3A OLCI data using the C2RCC with locally adapted parameters and compared to in-water Secchi depth measurements.

The proxy for Secchi depth derived using C2RCC-SNAP—“kd_z90 max” (Depth of the water column from which 90% of the water-leaving irradiance comes from) compared to in situ Secchi depth (SD) measured using Secchi disk, proved to have the lowest bias of −31% MNB, and relative error 46% RMSE and 78% APD, indicating that the satellite-derived proxy underestimates in-water Secchi depth measurements (Figure 7). Additionally, using the Secchi depth algorithm (Table 5) described in Alikas et al. [24] based on Kratzer et al. [34], which was developed using in-water data from Himmerfjärden bay and Pärnu bay (NE Gulf of Riga) and applied to the *K_d_*(489) (diffuse attenuation coefficient at 489 nm) output from the C2RCC-SNAP. The applied SD algorithm performed similar as the “kd_z90max” product, however only slightly overestimating Secchi depth with 6.4% MNB, 62% RMSE and 60% APD and with 10% lower spread (RMSE, relative error) and having the largest number of observations (*n* = 35) when compared to TSM, Chl-a and CDOM. 

**Figure 8:**
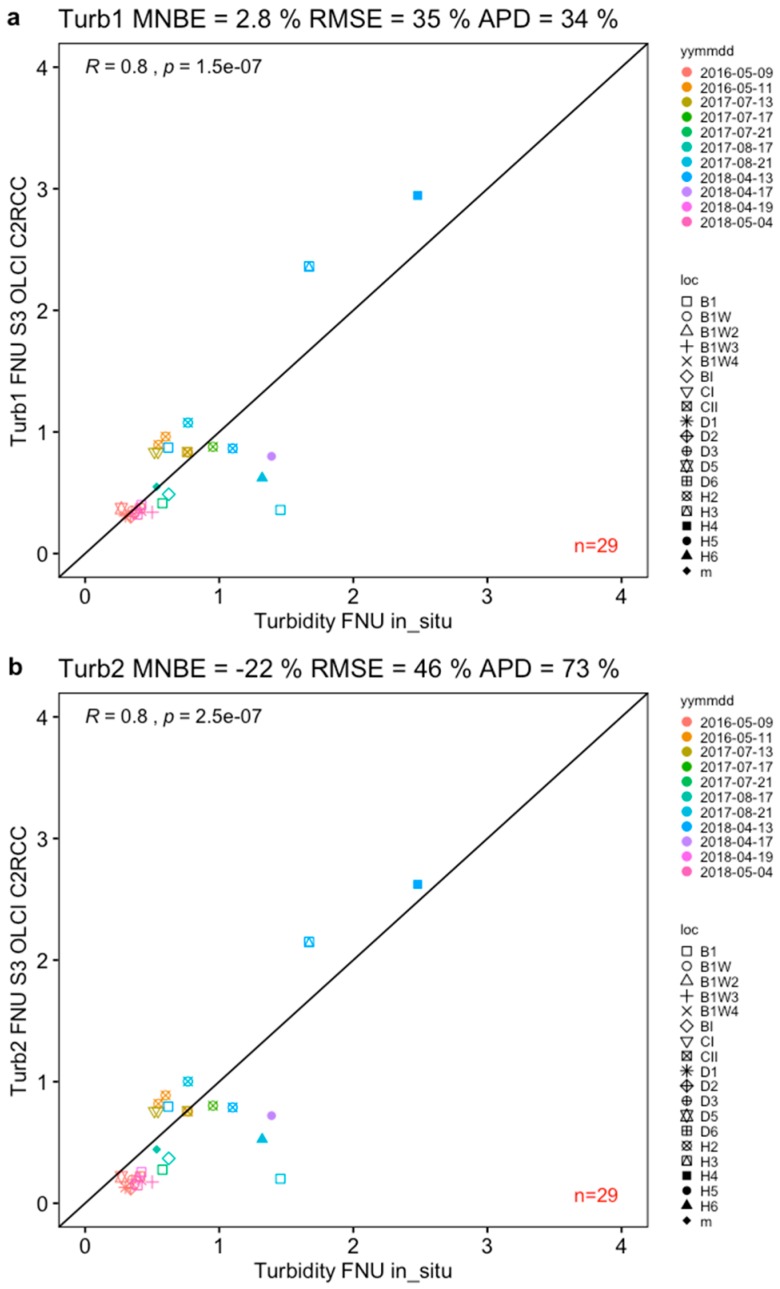
Turbidity products (**a**) Turb1 and (**b**) Turb2 derived from “iop_bpart” using the algorithms described above and applied to S3A OLCI data generated by the C2RCC-SNAP with locally adapted parameters and both compared to in situ turbidity.

The first turbidity algorithm (Turb1) applied to “iop_bpart” generated by C2RCC-SNAP - using the local in-water relationship between scatter measured by AC9 and turbidity measured by the bench turbidity meter-gave the lowest bias among other sets of comparisons (Figure 8a), with slight overestimations of only 2.8% MNB, 35% RMSE (relative error) and 34% APD (*n* = 29). The second algorithm (Turb2) based on ln-transformed regional data (including Bråviken and the Gulf of Bothnia) and applied to “iop_bpart” showed a nearly identical shape and a distribution of points with minor shifts. However, it gave an underestimation of −22% MNB, and a relative error of 46% (RMSE) and 73% APD (Figure 8b). The evaluation of the two algorithms indicates that the development of local relationships between optical and water quality measurements when applied to specific inherent optical properties may yield a reliable satellite-derived water product of relatively low bias and error. 

## 4. Discussion

### 4.1. Remote Sensing Reflectance

The remote sensing reflectance generated from Sentinel-3A OLCI data using C2RCC-SNAP processor were within similar ranges when compared to previous validation results for MERIS FR data in the NW Baltic proper [35,36]. However, an additional peak was detected at 490 nm (Figure 4) at sampling stations with high Secchi depth and low turbidity that were located closer to the coast and may also be influenced by adjacency effects from land. A similar unconventional peak was also shown in some of the OLCI reflectance spectra measured by Toming et al. [53] in Baltic Sea coastal waters in Estonia. However, a reflectance peak at 490 nm is usually only found in optical Case-1 waters, i.e., clear ocean waters, where the main optical component determining reflectance is phytoplankton with co-varying CDOM [42,54]. The Baltic Sea is comprised of optical Case-2 waters that are dominated by CDOM absorption [36,41,55,56], and with the reflectance peak typically found at 560 nm [35,36,57], which was also the case for the in situ stations sampled during the S3A OLCI validation campaigns in 2018. The observed peak at 490 nm in some of the early C2RCC-SNAP reflectance data may indicate an overcompensation of the atmospheric correction in the blue channels in near-coastal areas. 

Kratzer et al. [35] showed that the previous version of the C2R also had a problem with retrieving the correct spectral shape in the blue as the atmospheric correction seemed to overcorrect the atmospheric influence. It is possible that the atmospheric correction model applied in C2RCC [21,22] may also overcompensate for the negative reflectances that often occur in the blue part of the spectrum in waters with relatively high CDOM absorption [58]. A shift in the peak reflectance was also observed in the reflectance data from 9 May 2016. This could also be associated with discrepancies involving scenes captured by S3A OLCI during the commissioning period where sets of adjustments, sensor calibration, tests and satellite maneuvers were performed before the data passed quality control and became available to the user community. The scene from 9 May 2016 was one of the earliest images captured by S3A OLCI since its launch and has subsequently undergone a thorough reprocessing procedure (v2.23), however, might contain abnormally high reflectance values in the blue, or errors that may be associated with radiometric calibration [46]. Hence, scenes from the commissioning period should be treated with caution. 

The in situ reflectance data shown in Figure 4b indicates that such a peak in the blue does not actually occur in these waters with high CDOM absorption. At very high CDOM absorption as observed at some of the stations in the Bothnian Sea, the remote sensing reflectance is generally reduced and in this case the peak may even slightly be shifted towards the red end of the visible spectrum (not shown here). Generally, however, the reflectance signature peaks at 560 nm which is also in accordance with previous MERIS validation campaigns performed by other groups in the Baltic Sea area and other optically complex water bodies [59,60,61].

Besides problems with atmospheric correction, pixels close to land are also influenced by adjacency effects [62]. Kratzer et al. [35] and Beltran et al. [36] have found that the Improved Contrast between Ocean and Land processor (ICOL) developed by Santer et al. [62] has shown to improve retieval of remote sensing reflectance derived from MERIS data in near-coastal areas. Another approach to correct for the adjacency effect in optically-complex waters is the SIMilarity Environment Correction (SIMEC), which has also previously been applied to MERIS data with potential application to Sentinel-2, Sentinel-3, HyspIRI, EnMAP and PRISMA [63]. For OLCI, however, there are to date no algorithms available to correct for adjacency effects.

### 4.2. Level-2 Water Quality Products C2RCC-SNAP

This study aimed to improve the *TSM_NN* (i.e., Total Suspended Matter derived from the neural net) product from the OLCI ground segment by regionally adjusting the TSM-specific scatter, and some additional user-defined variables such as salinity and temperature. The regionally adjusted C2RCC-SNAP processor overestimated TSM with only 25% bias, which is an improvement compared to the *TSM_NN* standard product derived by the C2RCC EUMETSAT ground segment (L2W). In fact, the regionally adapted product shows similar reliability as MERIS data processed with the Case-2 Water Properties Processor developed by FUB (Free University Berlin [64,65]), which, however, underestimated TSM by about 27% [36], but so far has been the most reliable processor for Baltic Sea waters overall [35,36]. With regards to TSM retrieval, the C2RCC-SNAP performed here in accordance with its predecessor—C2R that has been tested on MERIS (only 5.5% and 25.2% bias in a pairwise comparison of different algorithms vs. in situ data) and further described in [36]. Chlorophyll a was retrieved in this study with only 21% bias, which is also within the error seen when applying the FUB processor to MERIS data (18%–27% MNB), but with substantial improvement compared to the C2R (above 104% MNB) [36]. An additional observation is that C2RCC-SNAP is not associated with significant noise that was present in MEGS (ground-segment implementation of C2R in MERIS) when deriving TSM and Chl-a. However, the C2RCC systematically underestimates CDOM when compared to the absorption coefficient of CDOM (Gelbstoff) at 443 nm (iop_agelb) (−41% MNB) and in a similar manner to previous versions of MEGS and C2R underestimated compared to in situ values during MERIS validation campaigns in 2008 and 2010 [35,36]. However, for S3A OLCI the bias was substantially reduced compared to MERIS (−68.3% to −90% MNB) [36], suggesting crucial improvements in the NN that computes absorption (from which CDOM is derived) in the C2RCC, even though the number of CDOM match-ups in this study was relatively low (*n* = 18) compared to other sets of water product comparisons. However, the C2RCC outputs several additional IOP’s, the absorption coefficient of detritus at 443 nm (iop_det), when summed up with “iop_agelb” forms an IOP denoted “iop_adg” (detritus + Gelbstoff absorption). When comparing the combined product “iop_adg” to in situ CDOM absorption there is even a lower bias of only 7.7% MNB, a similar spread of 56% RMSE and improved absolute percentage difference of 54%. We therefore would like to recommend the use of “iop_adg” for estimating CDOM in the Baltic Sea. Nevertheless, CDOM retrieval still remains a challenge, due to low performance of neural nets dealing with decreased reflectance, especially in the blue, in waters dominated by CDOM absorption such as the Baltic Sea [19], whilst the pigment absorption has a relatively small influence on the spectral absorption [41,66,67] and therefore also on the reflectance spectrum. Applying a previous version (v1.0) of the Case2 Regional algorithm (C2R) to MERIS data [19,68], Heim et al. [69] found that the main proportion of total absorption goes towards pigment absorption when using C2R in optically-complex waters, leading to an overestimation of Chl-a at low concentrations, and hence to a clear underestimation of aCDOM. This in turn affects the retrieval of TSM indirectly, as TSM (as mentioned above) is derived from scatter at 442 nm in an iterative fitting procedure to the remote sensing reflectance data (Schiller and Doerffer, 2006 & 2007) whilst CDOM and Chl-a absorption are jointly derived from MERIS absorption at 442 nm in the same iterative fitting procedure. 

The evaluation of the C2RCC in this paper and also in Plowey [70] shows that this problem seems to have been solved to some extent in the current C2RCC, providing rather reliable products for both Chl-a and CDOM in these concentration ranges, although, there is a clear underestimation of Chl-a at higher concentrations. This has also been observed in other studies using OLCI data above optically-complex lake waters [71,72]. The results presented in this current study show that when applying the regional TSM-specific scatter for the Baltic Sea [23] the retrieval of TSM becomes also reliable, especially when compared to the current standard processor for OLCI which showed extremely high errors for TSM in the same area of investigation [70]. Using a combination of ship monitoring data, data from an oceanographic mooring deployed at B1, and data from dedicated sea-truthing campaigns presented here, Plowey [70] also found that the TSM retrieval by the standard C2RCC NN product provided by EUMETSAT had rather high errors. However, the study by Plowey [70] also showed good statistics for *CHL_NN* (i.e., EUMETSAT standard product), predicting Chl-a concentrations with relatively low errors (MNB = −7%, RMSE = 40%, *n* = 156). Again, it was found that Chl-a was clearly underestimated at higher values. Besides a fault in the processor to accurately predict high Chl-a values at high CDOM absorption, this may also have been affected by adjacency effects from land in the inner bay, influencing the prediction of particle scatter, and subsequently also leading to inaccurate absorption estimates due to the iterative fitting method of the neural net and joint retrieval of absorption and scattering from a given remote sensing reflectance spectrum. Subsequently, the C2RCC-SNAP inversion algorithm may lead also to erroneous Chl-a concentration in coastal areas. However, turbidity—which was retrieved in Plowey [70] from *TSM_NN* using a reliable local algorithm [43]—showed extremely high errors (MNB 189%, RMSE 1011%, *n* = 45) when using the current standard C2RCC neural net (distributed by EUMETSAT) algorithm implemented in OLCI. CDOM—using *ADG_443_NN* as a proxy—also showed very good results, being only slightly underestimated (MNB −5%, RMSE = 37%, *n* = 36). 

The evaluation of the OLCI standard products for this campaign gave similar statistics. When comparing C2RCC-SNAP with the standard processor assessed with optical in situ data, Chl-a concentrations were retrieved with a relatively low systematic error (MNB = 10%) but a high relative error (RMSE = 97%, APD = 108%, *n* = 26) and, again, with a clear underestimation at higher values as was observed for the C2RCC-SNAP processor. TSM (*TSM_NN*) also showed a large systematic error (MNB: 103%), and an even larger relative error (RMSE: 167%), and an APD of 112%, *n* = 26. The CDOM (*ADG_443_NN* product) had a MNB of 85% and a very high relative error (RMSE of 220%), and an APD of 110% (*n* = 21). As mentioned before, the C2RCC-SNAP data were not vicariously calibrated, which is recommended by EUMETSAT. The lack of vicarious calibration may affect the statistics for the in-water products. However, System Vicarious Calibration (SVC) is always specific to the sensor and the applied atmospheric correction procedure. The currently SVC applied in the standard product was made for OLCI-A and standard Case-1 atmospheric correction. Currently, there is no publicly available SVC for OLCI-A and C2RCC and there is no general consensus whether vicarious gains improve the retrieval of water products in the Baltic Sea.

In summary, the standard product for TSM provided by EUMETSAT shows very high systematic errors, while Chl-a seems to be retrieved rather well at concentrations below 10 µgL^−1^. Note that the cloud masking using recommended flags for the standard product [13] differs to C2RCC [18], and the errors are therefore not directly comparable, and both products also retrieve slightly different numbers of observation per product.

Secchi depth can be derived from the spectral attenuation coefficient *K_d_*(490) using the empirical relationship between *K_d_*(490) and in situ Secchi depth as described in [24,34,73]). The development of *K_d_*(490) algorithms goes way back to the SeaWiFS sensor launched by NASA in 1997 [73,74,75]. *K_d_*(490) is strongly correlated to Secchi depth [73] whilst Secchi depth is one of the most robust water products derived in the Baltic Sea. It has also been evaluated for use in integrated coastal zone management [76]. Similar algorithms are also being developed and applied to inland and other coastal waters, allowing to generate Secchi depth time-series over large spatial and temporal scales [24]. These water products can then be evaluated from a eutrophication and management perspective [52,76]. Amongst the C2RCC-SNAP L2 products, the closest proxy to in-water Secchi depth is the product “kd_z90max”, which is derived from the minimum diffuse attenuation, *K_d_*(min). i.e., the mean downwelling attenuation coefficient of the three bands with minimum *K_d_*. “kd_z90max” is given in the unit of meter (m) which makes it possible to compare it directly to in situ Secchi depth, although the Secchi depth is measured using the full visible spectrum. Unpublished experiments from the marine remote sensing group at Stockholm University using Goggles fitted with green (spectral) interference filters whilst measuring the Secchi depth in the Baltic Sea have shown, that the green light gives the same Secchi depths as a Secchi depth reading taken without any interference filters. This can be explained by the green transmission peak in Baltic Sea waters at about 560 nm [77], which in turn explains the reflectance peak at 560 nm (Figure 4b).

The “kd_z90max” product derived from S3A OLCI performed reasonably well in this study when compared to Secchi depth with an underestimation of 31% MNB. The applied regional algorithm developed by Alikas et al. [24] that allows to derived Secchi depth from *K_d_*(490), however, performed substantially better in terms of bias (6.4% MNB), but with an increased relative error of 62% RMSE, though slightly improved APD of 60%. This approach thus is capable of providing users with a reliable water quality product derived via various outputs from the C2RCC-SNAP processor that can be used for assessing water clarity as well as used as an indicator for eutrophication [78,79]. Secchi depth is also relatively easy to measure compared to more laborious procedures associated with measuring Chl-a absorption and gravimetric measurements of TSM, thus leading to a higher number of match-ups in this study (*n* = 35), especially when using small boats for validation. Secchi depth can also be modelled using a multiple regression approach [80].

One of the most interesting results that came out from applying C2RCC-SNAP to S3A OLCI data and comparing the results to in situ measurements was how well turbidity—modelled from “iop_bpart” using the local in-water relationship (Turb1)—compared to turbidity measured using a bench turbidity meter (with only 2.8% MNB). The algorithm was very similar to the regional algorithm derived from a larger data set covering a large range of optical conditions found in the Baltic Sea region, including coastal areas in the Bothnia Sea and also Bråviken bay. The ln-transformed version of the same algorithm (Turb2) performed somewhat less well with −22% MNB and higher spread 46% RMSE and reduced APD of 73%, however when visually examining the scatter of the data points on the graph (Figure 8), the positioning of the points differ almost negligibly to Turb1, suggesting that the metrics used in the evaluation e.g., MNB, RMSE and APD should be used with caution, since even slightest shift in the relative scatter can produce a significant difference (>5%) in error. As the local turbidity-particle scatter algorithm is very similar to the non-linear form of the more regionally-derived algorithm (including data from Bråviken bay and even the Bothnian Sea), we would like to suggest the use of either of these two linear algorithms for the Baltic Sea region. Future validation campaigns in highly absorbing waters will be performed in order to further test and validate these turbidity algorithms.

The C2RCC-SNAP processor allows to generate IOP’s (i.e., CDOM absorption and particle scatter) as products. In the MERIS processor IOP’s were only used in an intermittent step within the processing chain to compute the final water products Chl-a, TSM and CDOM from absorption and scattering at 442 nm. The architecture of the C2RCC-SNAP makes it easier to modify the conversion factors between IOP’s and concentrations in order to derive the water products directly from the IOP product using the specific IOP’s (i.e., the regional conversion factors).

Another option is to derived turbidity from TSM [43] as their close relationship allows for a very robust empirical algorithm between turbidity and TSM measured in situ that can subsequently be applied to the TSM satellite product “conc_tsm” in order to generate turbidity from satellite data, and consequently to map turbidity over large areas [43]. There are also algorithms to derive turbidity from single band reflectance in the red or NIR in waters with high TSM loads [81]. However, these algorithms have shown not to be applicable to Baltic Sea and other CDOM-dominated waters [43,82]. The approach presented here—i.e., deriving turbidity from the remote sensing TSM product-seemed to work well in the Baltic. However, the generated turbidity algorithm presented in Kari et al. [43] had not been validated against in situ measurements, although the derived values were found to be within the typical turbidity and TSM ranges previously observed in the NW Baltic proper. 

In this current study, we applied a local in-water relationship between scatter at 440 nm (*b*_440_) and turbidity to satellite-derived “iop_bpart” which then generated a turbidity product (Turb1 and Turb2) directly from particle scatter. This product was subsequently validated against an independent in situ dataset (Turb1). This approach allows for potentially more accurate retrieval of turbidity data directly from the particle scatter at 442 nm derived from remote sensing reflectance without going through the empirical relationship between TSM and turbidity as proposed in [43]. 

Turbidity is also interesting from a management perspective as it is one of the key parameters emphasized in the communication of the Marine Strategy Framework Directive [83]. Annex III of this directive lists turbidity as one of the mandatory physical and chemical parameters to be measured in coastal waters. Remote sensing is a cost-effective method to do so as it has improved temporal and spatial coverage when compared to conventional monitoring data [84,85] despite the frequent cloud coverage in the Baltic Sea.

## 5. Conclusions

The C2RCC-SNAP has been applied to Sentinel-3A OLCI L1 data using SNAP and validated against dedicated in situ data in the Northwestern Baltic proper (Himmerfjärden and surrounding open sea areas, including Landsort Deep) collected during several dedicated OLCI validation campaigns. The validation campaigns took place between 2016 and 2018 in Swedish coastal waters and covered different times of year, including the cyanobacteria blooms in summer 2017 and the development of the spring bloom in 2018. The campaigns covered nearshore - open sea transects with strong optical gradients and a reasonable number of good quality match-up stations. 

Overall, the C2RCC-SNAP performed well in retrieving remote sensing spectrum with a typical shape for Baltic Sea waters with the reflectance peaking at 560 nm. However, at some of the near-coastal stations with low turbidity (0.27–0.57 FNU) and high Secchi depth (5.5–11 m) an erroneous peak was observed at 490 nm. This peak is likely to be an artefact and maybe caused by an over-shoot of the atmospheric correction in the blue channels.

The retrieval of water quality products and its consecutive validation, was overall successful with rather reliable results (relatively low bias and scatter) for Chl-a, TSM, CDOM and Secchi depth (kd_z90max) and comparing well to the retrieval of L2W products by the MERIS standard processor MEGS (v3). This should be seen as a clear success of the ESA/EUMETSAT OLCI mission—after only a few years of operation in Space. In comparison, it took many years into the MERIS mission to achieve the same quality of water product retrieval from optically-complex waters. The success of the OLCI mission, in turn, is clearly based on prior experience and lessons learnt from MERIS. More work needs to be done in order to correct OLCI data for adjacency effects from land.

The retrieval of TSM using the regional TSM-specific scatter gave very good results with very low bias, allowing for substantially improved retrieval of TSM, when compared to the current S3A OLCI EUMETSAT ground segment standard processor. A local algorithm (Turb1) to derive turbidity from particle scatter “iop_bpart” was developed (using data from 2000–2010) and showed good results against an independent data set (from 2016–2018).

We recommend the use of the C2RCC-SNAP with regionally adjusted TSM-specific scatter for L2 water product retrieval as well as the regional turbidity algorithm for Baltic Sea monitoring which is a good tool for management of coastal waters. Additional validation work is planned to increase the number of match-ups and to evaluate the regional and local turbidity algorithms presented in the study. The reflectance data measured by OLCI mounted on S3A and S3B in the Gulf of Bothnia is currently assessed against the measured in situ reflectance data, comparing several available OLCI processors against data measured in situ. This will allow for rigorous assessment of OLCI L2 products also in water with extremely high CDOM absorption.

## Figures and Tables

**Table 1:** OLCI bands with MERIS heritage bands and additional highlighted in bold (source: ESA).

Band i.d.	Center Wavelength (nm)	Bandwidth (nm)
**Oa1**	400	15
Oa2	412.5	10
Oa3	442.5	10
Oa4	490	10
Oa5	510	10
Oa6	560	10
Oa7	620	10
Oa8	665	10
**Oa9**	673.75	7.5
Oa10	681.25	7.5
Oa11	708.75	10
Oa12	753.75	7.5
Oa13	761.25	2.5
**Oa14**	764.375	3.75
**Oa15**	767.5	2.5
Oa16	778.75	15
Oa17	865	20
Oa18	885	10
Oa19	900	10
**Oa20**	940	20
**Oa21**	1020	40

**Table 2:** List of optical stations during Sentinel-3A OLCI validation campaigns 2016–2018 in the NW Baltic proper. * Indicates the time lag between the in situ sampling and the S3 overpass. ** Indicates that the match-up station was under cloud.

Time [UTC + 0]				Sentinel-3 Matchup Window				
Cast ID	Date	*In situ*	Overpass Start	Cloudy	<30 min	≤1 h	≤2 h	>2 h
D0_a	9 May 2016	09:39:00	08:58:42			*		
D1_b		10:04:00	08:58:42				*	
D2_c		10:18:00	08:58:42				*	
D3_d		10:32:00	08:58:42				*	
D4_f		10:45:00	08:58:42				*	
D5_f		10:57:00	08:58:42				*	
D6_g		11:09:00	08:58:42					*
B1_h		05:20:00	08:58:42				*	
H2_i		06:42:00	08:58:42		*			
H3_j		09:50:00	08:58:42			*		
H4_k		07:35:00	08:58:42				*	
H5_l		08:10:00	08:58:42			*		
H6_m		08:30:00	08:58:42		*			
H2_a	11 May 2016	09:09:00	09:47:19	**		*		
H2_b		09:39:00	09:47:19	**	*			
H2_e		10:17:00	09:47:19	**	*			
CII_a	11 May 2016	08:58:00	09:21:07		*			
CII_c		09:57:00	09:21:07			*		
CII_e		11:20:00	09:21:07			*		
CII_1a	13 July 2017	08:45:00	09:51:11				*	
CI_1b		x	09:51:11					
CI_1c		x	09:51:11					
H2_2a	17 July 2017	08:25:00	09:47:27				*	
H4_2b		10:15:00	09:47:27		*			
H3_2c		11:10:00	09:47:27				*	
H5_3a	21 July 2017	08:30:00	09:43:42	**			*	
H2_3b		10:12:00	09:43:42		*			
B1_3c		11:15:00	09:43:42	**			*	
BIII_4a	9 Aug. 2017	08:25:00	09:51:10	**			*	
BII_4b		10:45:00	09:51:10	**		*		
B1_4c		12:45:00	09:51:10	**				*
BI_5a	17 Aug. 2017	07:15:00	09:43:40					*
m_5b		07:40:00	09:43:40					*
m_5c		08:05:00	09:43:40				*	
m_5d		08:35:00	09:43:40				*	
B1_6a	21 Aug. 2017	05:45:00	09:39:55					*
H2_6b		07:10:00	09:39:55					*
H3_6c		09:08:00	09:39:55			*		
H4_6d		08:15:00	09:39:55				*	
H5_6e		11:36:00	09:39:55				*	
H6_6f		x	09:39:55					
B1_7a	22 Aug. 2017	07:10:00	09:13:42					*
H3_7b		08:15:00	09:13:42	**		*		
H4_7c		08:45:00	09:13:42	**	*			
BII_1a	9 April 2018	08:30:00	09:51:11	**			*	
BIS_1b		09:50:00	09:51:11	**	*			
B1_1c		11:10:00	09:51:11	**			*	
H4_2a	13 April 2018	08:03:00	09:47:26				*	
H3_2b		09:17:00	09:47:26		*			
H2_2c		10:57:00	09:47:26				*	
B1_2d		11:55:00	09:47:26					*
B1_3a	17 April 2018	06:20:00	09:43:42	**				*
H3_3b		08:55:00	09:43:42			*		
H4_3c		08:05:00	09:43:42				*	
H5_3d		10:52:00	09:43:42				*	
H6_3e		10:20:00	09:43:42			*		
H4_4a	19 April 2018	08:10:00	08:51:20			*		
H5_4b		09:22:00	08:51:20		*			
H3_4c		11:35:00	08:51:20					*
B1_4d		13:30:00	08:51:20					*
B1_7a	4 May 2018	08:45:00	09:02:33		*			
B1W_7b		09:01:00	09:02:33		*			
B1W2_7c		09:10:00	09:02:33		*			
B1W3_7d		09:19:00	09:02:33		*			
B1W4_7e		09:34:00	09:02:33			*		

**Table 3:** List of Level-1b products used for the match-up analysis.

List of Level-1 Full Resolution OLCI Products	Products Availability
S3A_OL_1_EFR____20160509T085842_20160509T090042_20170929T065132_0119_004_050______MR1_R_NT_002.SEN3	CODArep (https://codarep.eumetsat.int)
S3A_OL_1_EFR____20160511T094719_20160511T094919_20170929T091509_0119_004_079______MR1_R_NT_002.SEN3	
S3A_OL_1_EFR____20160512T092107_20160512T092307_20170929T102634_0119_004_093______MR1_R_NT_002.SEN3	
S3A_OL_1_EFR____20170713T095111_20170713T095311_20171021T102121_0119_020_022______MR1_R_NT_002.SEN3	
S3A_OL_1_EFR____20170717T094727_20170717T094927_20171021T171336_0119_020_079______MR1_R_NT_002.SEN3	
S3A_OL_1_EFR____20170721T094342_20170721T094542_20171022T000942_0119_020_136______MR1_R_NT_002.SEN3	
S3A_OL_1_EFR____20170809T095110_20170809T095310_20171216T030232_0119_021_022______MR1_R_NT_002.SEN3	
S3A_OL_1_EFR____20170817T094340_20170817T094540_20171216T125353_0119_021_136______MR1_R_NT_002.SEN3	
S3A_OL_1_EFR____20170821T093955_20170821T094155_20171216T174456_0119_021_193______MR1_R_NT_002.SEN3	
S3A_OL_1_EFR____20170822T091342_20170822T091542_20171216T185333_0119_021_207______MR1_R_NT_002.SEN3	
S3A_OL_1_EFR____20180409T095111_20180409T095411_20180410T153428_0179_030_022_1980_MAR_O_NT_002.SEN3	CODA (https://coda.eumetsat.int)
S3A_OL_1_EFR____20180413T094726_20180413T095026_20180414T160621_0179_030_079_1980_MAR_O_NT_002.SEN3	
S3A_OL_1_EFR____20180417T094342_20180417T094642_20180418T152141_0180_030_136_1980_MAR_O_NT_002.SEN3	
S3A_OL_1_EFR____20180419T085120_20180419T085420_20180420T141404_0179_030_164_1980_MAR_O_NT_002.SEN3	
S3A_OL_1_EFR____20180504T090233_20180504T090533_20180505T140824_0179_030_378_1980_MAR_O_NT_002.SEN3	

**Table 4:** C2RCC OLCI processing parameters used for processing match-up scenes during validation campaigns 2016–2018. * indicates parameters that are automatically imported from L1b data and used for processing. Values listed in bold were adjusted to specific Baltic Sea conditions. ** derived in ref. [23].

C2RCC OLCI Processing Parameters.			
Date	May 2016	July–August 2017	April–May 2018
Valid-pixel expression	default	default	default
Salinity	**7**	**7**	**7**
Temperature	**5**	**15**	**5**
Ozone	330 *	330 *	330 *
Air Pressure	1000 *	1000 *	1000 *
TSM factor bpart	0.986 **	0.986 **	0.986 **
TSM factor bwit	1.72	1.72	1.72
CHL exponent	1.04	1.04	1.04
CHL factor	21	21	21
Threshold rtosa OOS	0.05	0.05	0.05
Threshold AC reflectances OOS	0.1	0.1	0.1
Threshold for cloud flag on transmittance down @865	0.955	0.955	0.955
Atmospheric aux data path	default	default	default
Alternative NN Path	default	default	default
Output AC reflectances as *R_rs_* instead of rhow	On	On	On
Derive water reflectance from path radiance and transmittance	Off	Off	Off
Use ECMWF aux data of source product	On	On	On
Output TOA reflectances	On	On	On
Output gas corrected TOSA reflectances	Off	Off	Off
Output gas corrected TOSA reflectances of auto NN	Off	Off	Off
Output path radiance reflectances	Off	Off	Off
Output downward transmittance	Off	Off	Off
Output upward transmittance	Off	Off	Off
Output atmospherically corrected angular dependent reflectances	On	On	On
Output normalized water-leaving reflectances	On	On	On
Output of out of scope values	Off	Off	Off
Output of irradiance attenuation coefficients	On	On	On
Output uncertainties	On	On	On

**Table 5:** The output L2 products generated by C2RCC [18]. The products in bold were validated in this paper.

Output L2 Products Generated by C2RCC		
Product Name	Description	Unit
Rtoa 400–1020 nm	Top-of-atmosphere reflectance	
***Rrs* 400–1020 nm**	Atmospherically corrected angular dependent remote sensing reflectances	sr^−1^
Rhow 400–1020 nm	Atmospherically corrected angular dependent water-leaving reflectances, Rhow = *Rrs* × π	
Diffuse attenuation coefficicent		
***kd*489**	Irradiance attenuation coefficient at 489 nm	m^−1^
*kd*min	Mean irradiance attenuation coefficient at the three bands with minimum *kd*	m^−1^
**kd_z90max**	Depth of the water column from which 90% of the water-leaving irradiance comes from (1/*kdmin*)	m
Inherent optical properties		
iop_apig	Absorption coefficient of phytoplankton pigments at 443 nm	m^−1^
iop_adet	Absorption coefficient of detritus at 443 nm	m^−1^
**iop_agelb**	Absorption coefficient of Gelbstoff at 443 nm	m^−1^
iop_bpart	Scattering coefficient of marine particles at 443 nm	m^−1^
iop_bwit	Scattering coefficient of white particles at 443 nm	m^−1^
**iop_adg**	Detritus + gelbstoff absorption at 443 nm (iop_adet + iop_agelb)	m^−1^
iop_atot	phytoplankton + detritus + gelbstoff absorption at 443 nm (iop_apig + iop_adet + iop_agelb)	m^−1^
iop_btot	total particle scattering at 443 nm (iop_bpart + iop_bwit)	m^−1^
Concentrations (conc)		
**conc_tsm**	Total suspended matter dry weight concentration (iop_bpart × 0.986 + iop_bwit × 1.72)	gm^−3^
**conc_chl**	Chlorophyll concentration (pow (iop_apig, 1.04) × 21.0)	µgL^−1^
User-defined		
**SD**	Secchi depth = 2.39 × (*kd*489^−0.86^) [24]	m
**Turb1**	Turbidity = 0.99 × iop_b_part_ + 0.24	FNU
**Turb2**	Turbidity = exp ((0.82 × ln (iop_b_part_) + 0.14)	FNU
